# Wet End Chemical Properties of a New Kind of Fire-Resistant Paper Pulp Based on Ultralong Hydroxyapatite Nanowires

**DOI:** 10.3390/molecules27206808

**Published:** 2022-10-11

**Authors:** Li-Ying Dong, Ying-Jie Zhu, Jin Wu

**Affiliations:** 1 State Key Laboratory of High Performance Ceramics and Superfine Microstructure, Shanghai Institute of Ceramics, Chinese Academy of Sciences, Shanghai 200050, China; 2 Center of Materials Science and Optoelectronics Engineering, University of Chinese Academy of Sciences, Beijing 100049, China

**Keywords:** nanowire, hydroxyapatite, fire-resistant paper, paper pulp, wet end chemical properties, paper making

## Abstract

In 2014, a new type of the fire-resistant paper based on ultralong hydroxyapatite (HAP) nanowires was reported by the author’s research group, which had superior properties and promising applications in various fields, such as high-temperature resistance, fire retardance, heat insulation, electrical insulation, energy, environmental protection, and biomedicine. The wet end chemical properties of the fire-resistant paper pulp are very important for papermaking and mechanical performance of the paper, which play a guiding role in the practical production of the fire-resistant paper. In this paper, the wet end chemical properties of a new kind of fire-resistant paper pulp based on ultralong HAP nanowires are studied for the first time by focusing on the wet end chemical parameters, the effects of these parameters on the properties such as flocculation, retention, draining, and white water circulation of the fire-resistant paper pulp, and their effects on the properties of the as-prepared fire-resistant paper. The experimental results indicated that the wet end chemical properties of the new kind of fire-resistant paper pulp based on ultralong HAP nanowires were unique and entirely different from those of the traditional paper pulp based on plant fibers. The wet end chemical properties of the fire-resistant paper pulp were significantly influenced by the inorganic adhesive and its content, which affected the runnability of the paper machine and the properties of the as-prepared fire-resistant paper. The flocculation properties of the fire-resistant paper pulp based on ultralong HAP nanowires were affected by the conductivity and Zeta potential. The addition of the inorganic adhesive in the fire-resistant paper pulp based on ultralong HAP nanowires could significantly increase the conductivity of the fire-resistant paper pulp, reduce the particle size of paper pulp floccules, and increase the tensile strength of the fire-resistant paper. In addition, the fire-resistant paper pulp based on ultralong HAP nanowires in the presence of inorganic adhesive exhibited excellent antibacterial performance. This work will contribute to and accelerate the commercialization process and applications of the new type of the fire-resistant paper based on ultralong HAP nanowires.

## 1. Introduction

The wet end chemical properties and mechanisms of the paper pulp are important for paper making and the mechanical performance of paper. The studies on wet end chemical properties and mechanisms of the traditional paper pulp based on cellulose fibers are relatively mature, and a great volume of research work has been done [1,2,3,4,5,6], which play a guiding role in the practical production of the traditional paper based on cellulose fibers.

Hydroxyapatite (Ca_10_(PO_4_)_6_(OH)_2_, HAP) is an important kind of biomaterial especially in biology since it is the main inorganic constituent of bone and tooth [7]. HAP has a high biocompatibility, high whiteness, high-temperature resistance, and non-flammability [8,9]. Unfortunately, traditional hydroxyapatite materials are usually as hard and brittle as tooth and bone, and are difficult to be used for making soft functional materials [10]. Among various morphologies of HAP, such as particles, rods, sheets, needles, and wires, HAP nanowires are promising for applications in various fields. However, the research on HAP nanowires was less reported in the literature due to the difficulty in the synthesis of HAP nanowires. Only a few synthetic methods were reported for the synthesis of HAP nanowires, including the solvothermal/hydrothermal method [11,12,13], microwave-assisted synthesis [14], hard template method [15,16], sol-gel hydrothermal method [17], and reverse micelle hydrothermal method [18]. However, the lengths of the reported HAP nanowires are usually short (<10 µm), and these HAP nanowires with relatively small aspect ratios are still brittle and not highly flexible. The synthesis of highly flexible ultralong HAP nanowires with nanoscale diameters and ultrahigh aspect ratios is still a great challenge [19,20].

Ultralong HAP nanowires are defined as HAP nanowires with diameters of <100 nm, lengths of >100 µm, and aspect ratios of >1000 [19]. Ultralong HAP nanowires have several advantages such as high biocompatibility, high flexibility, high whiteness, tunable surface hydrophilicity/hydrophobicity, abundant surface functional groups, nonflammability, and high thermal stability. As a result, ultralong HAP nanowires are the ideal building blocks for the construction of various high-performance functional materials, and they are promising for applications in various fields such as high-temperature resistance, fire retardance, heat insulation, electrical insulation, energy, environmental protection, and biomedicine [19,20]

In 2014, the author’s research group reported the calcium oleate precursor solvothermal method for the synthesis of highly flexible ultralong HAP nanowires [21]. Ultralong HAP nanowires with diameters of ~10 nm and lengths of several hundred micrometers and aspect ratios of >10,000 were prepared by this method [22,23,24]. The nanoscale diameter, ultralong length and ultrahigh aspect ratio of the as-prepared ultralong HAP nanowires resulted in high flexibility, which could solve the problems of high brittleness and hardness of the traditional HAP materials. Ultralong HAP nanowires could be used as the raw material for making a new kind of highly flexible fire-resistant inorganic paper [21]. Later, the author’s research group further reported the calcium fatty acid (calcium oleate, calcium laurate, calcium stearate, etc.) precursor hydrothermal method for the synthesis of ultralong HAP nanowires using water as the only solvent in the absence of any organic solvent [25]. The author’s research group also developed the microwave-assisted calcium fatty acid precursor solvothermal/hydrothermal method for rapid synthesis (within 1 h) of ultralong HAP nanowires with advantages such as high efficiency, low cost, time saving, and energy saving [26].

In contrast to the flammable organic cellulose fibers as the raw material for the traditional paper making, ultralong HAP nanowires were nonflammable and high-temperature-resistant. It should be noted that inorganic fibers, such as aluminum silicate fibers, magnesium silicate fibers, alumina fibers, and glass fibers have no hydroxyl groups on the fiber surface. However, there are plenty of hydroxyl groups on the surface of ultralong HAP nanowires, which was the prerequisite for the formation of hydrogen bonds between ultralong HAP nanowires. Hydrogen bonding between fibers was one of the most important reasons for the high mechanical strength of the traditional paper based on plant fibers. In addition, ultralong HAP nanowires exhibited high specific surface area, high whiteness, and excellent heat and electrical insulation performance. Therefore, ultralong HAP nanowires were an ideal raw material for preparing a new kind of the fire-resistant paper, which had promising applications in various fields such as high-temperature resistance, fire retardance, heat insulation, electrical insulation, energy, environmental protection, and biomedicine. In recent years, a lot of research findings for ultralong HAP nanowires and their functional materials have been reported by the author’s research group [27,28,29,30,31,32,33,34,35,36,37,38,39,40,41,42].

The mass production technology of the new kind of the fire-resistant paper based on ultralong HAP nanowires is critical for its commercialization and large-scale applications. The wet end chemical properties of the new kind of fire-resistant paper pulp based on ultralong HAP nanowires are very important for the papermaking and properties of the fire-resistant paper, which is the theoretical basis for the mass production technology of the new kind of fire-resistant paper. However, the wet end chemical properties of the new kind of fire-resistant paper pulp based on ultralong HAP nanowires have not been investigated up to now, and there is an urgent need for the relevant research on this topic.

In this work, the wet end chemical properties of the new kind of fire-resistant paper pulp based on ultralong HAP nanowires are studied by focusing on the wet end chemical parameters, the effects of these parameters on the properties such as flocculation, retention, draining, molding, and white water circulation of the fire-resistant paper pulp, and the effects of wet end chemical parameters on the properties of the fire-resistant paper. The experimental results indicated that the wet end chemical properties of the new kind of fire-resistant paper pulp based on ultralong HAP nanowires were unique and totally different from those of the traditional paper pulp based on plant fibers. This work will provide the theoretical basis for the mass production technology of the new kind of fire-resistant paper and accelerate the commercialization and applications of the fire-resistant paper based on ultralong HAP nanowires.

## 2. Results and Discussion

### 2.1. Characterization of Ultralong HAP Nanowires and the Fire-Resistant Paper Pulp

**Figure 1:**
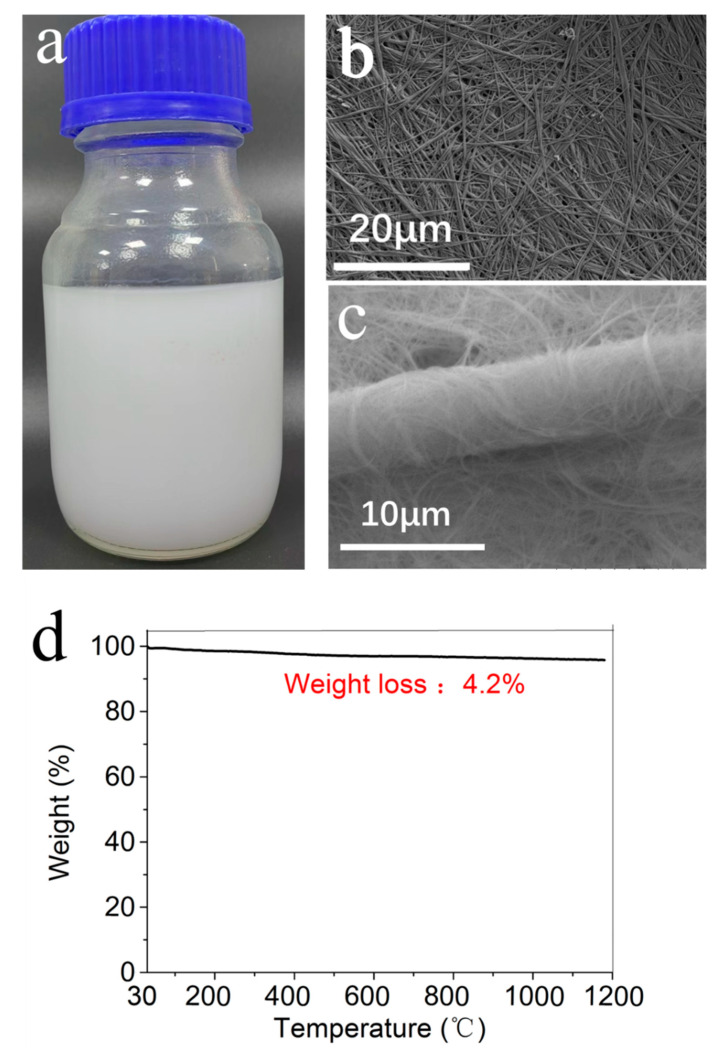
(**a**) A digital image of the fire-resistant paper pulp composed of ultralong HAP nanowires, micrometer-sized glass fibers, and nanometer-sized inorganic adhesive; (**b**) A SEM image of ultralong HAP nanowires; (**c**) A SEM image of the fire-resistant paper pulp composed of ultralong HAP nanowires and micrometer-sized glass fibers; (**d**) TG curve of ultralong HAP nanowires.

The fire-resistant paper pulp was a white aqueous suspension composed of ultralong HAP nanowires, micrometer-scale glass fibers, and nano-sized inorganic adhesive, as shown in Figure 1a. A SEM image of ultralong HAP nanowires is shown in Figure 1b, from which one can see that the as-prepared ultralong HAP nanowires had diameters of 10–20 nm and lengths of several hundred micrometers. In many cases, ultralong HAP nanowires self-assembled along the longitudinal direction to form nanowire bundles with larger diameters.

Figure 1c shows a SEM image of the fire-resistant paper pulp consisting of ultralong HAP nanowires and micrometer-sized glass fibers, and one can see that micrometer-sized glass fibers were wrapped with ultralong HAP nanowires, the interactions among ultralong HAP nanowires and micrometer-sized glass fibers resulted in enhanced mechanical strength. In addition, the abundant hydroxyl groups on the surface of ultralong HAP nanowires contributed to the formation of hydrogen bonds between ultralong HAP nanowires and glass fibers, thereby improving the mechanical strength of the fire-resistant paper. In order to further enhance the mechanical properties of the fire-resistant paper, the inorganic adhesive (IA) was added to the fire-resistant paper pulp.

The analysis of the TG curve indicated the high stability of ultralong HAP nanowires even at high temperatures up to 1200 °C, the weight loss of ultralong HAP nanowires was as small as 4.2% of the original weight in the temperature range of 30 °C to ~1200 °C. The small weight loss of ultralong HAP nanowires was attributed to the loss of the water and oleate groups adsorbed on the surface of ultralong HAP nanowires.

### 2.2. Laboratory Simulation Studies on Wet End Chemical Properties of the Fire-Resistant Paper Pulp Based on Ultralong HAP Nanowires

**Figure 2:**
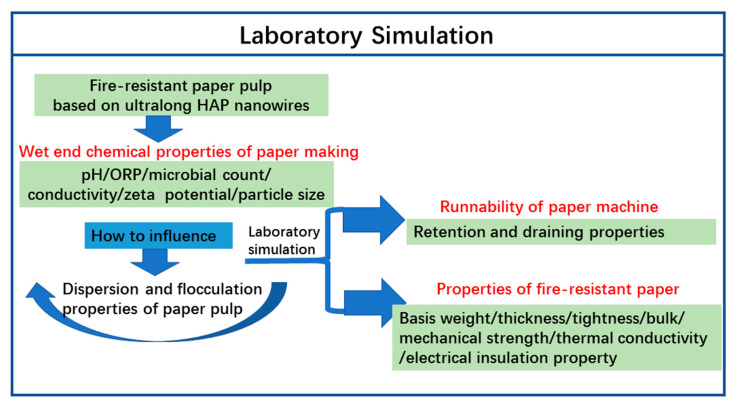
The schematic illustration for the laboratory simulation studies on wet end chemical properties of the fire-resistant paper pulp based on ultralong HAP nanowires.

The interaction and mechanism between paper components are closely related to the properties (size, specific surface area, surface functional groups, surface charge, etc.) of each component. The composition of the traditional paper pulp based on cellulose fibers is extremely complex, which contains large-sized plant fibers (the typical sizes of plant fibers are 1–3 mm in length and 10–20 µm in width), fiber fines (less than 76 µm in size), granular fillers (0.1–10 µm), colloidal particles, and soluble polymers. In contrast, the fire-resistant paper pulp contains ultralong HAP nanowires as the main fire-resistant material, micrometer-sized glass fibers (GFs) as the reinforcing skeleton material, and self-made nanoscale inorganic adhesive as a glue. There are several key issues which need to be researched, for example, how do these components in the fire-resistant paper pulp interact with each other? How do the wet end chemical parameters (pH value, conductivity, ORP, microbiological property, Zeta potential, particle size, etc.) affect the properties such as flocculation, retention, draining, molding, and white water circulation of the fire-resistant paper pulp? How do the wet end chemical parameters affect the runnability of the paper machine and the properties of the as-prepared fire-resistant paper? In this article, the above scientific questions are studied in depth through the laboratory simulation of the whole papermaking process of the fire-resistant paper (Figure 2).

### 2.3. Wet End Chemical Parameters and Their Influencing Factors

The essence of the paper making is the process of forming paper from the paper pulp through water filtration, press dehydration, and drying. A large amount of white water is removed in the wet end, which is recycled in the white water closed circulation system. In the traditional papermaking process, the microorganisms are easy to breed in the wet end, which causes the high microbial count in the white water closed circulation system. The reproduction of microorganisms will produce organic acids and polysaccharides, causing a decreased pH value and pollution of the wet end system. Furthermore, decaying paper pulp caused by the microbial growth can cause diseases. Therefore, it is important to inhibit the propagation of microorganisms in the process of paper making.

**Figure 3:**
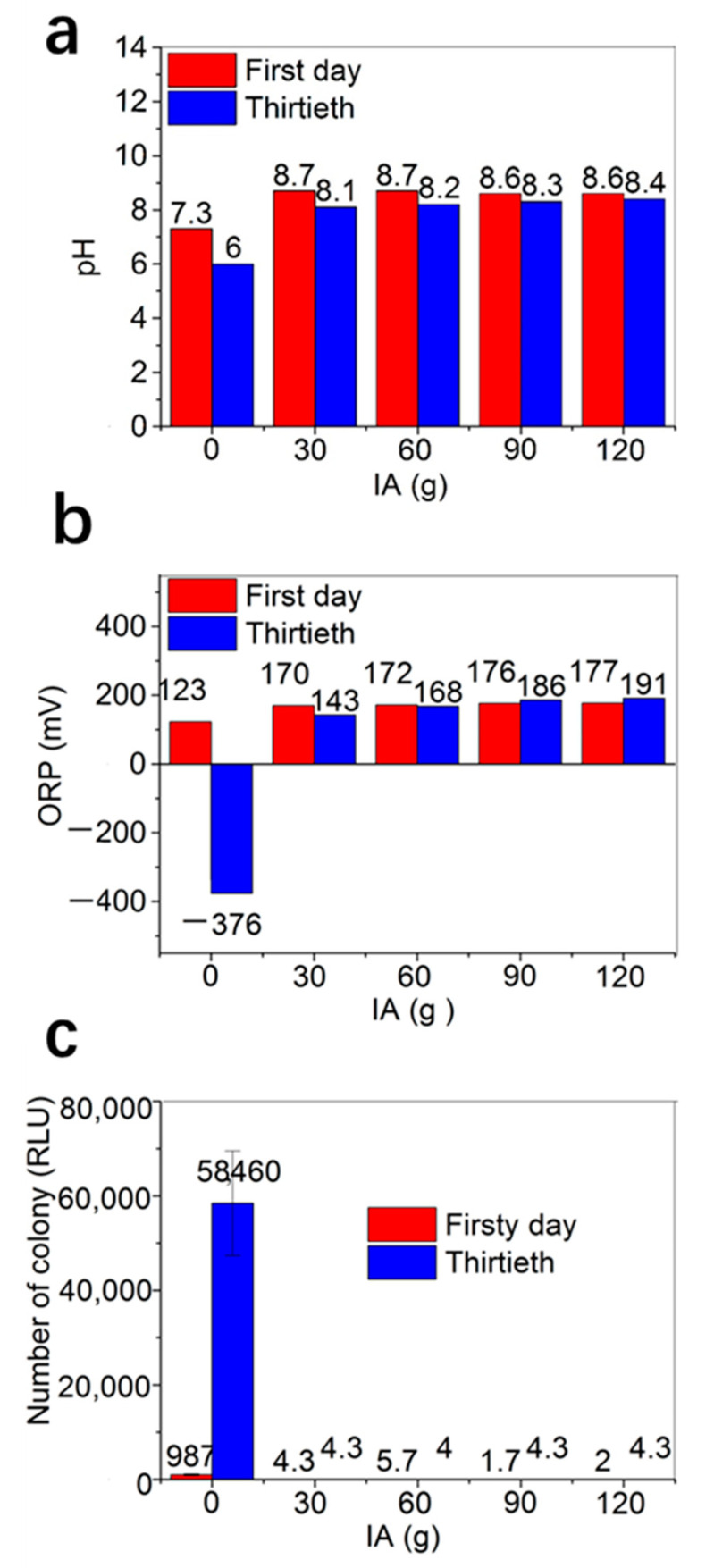
The pH values (**a**), ORP values (**b**), and numbers of bacterial colony (**c**) of the fire-resistant paper pulps with different amounts of IA stored at room temperature (28–36 °C) for 30 days in summer.

How about the propagation of microorganisms in the fire-resistant paper pulp based on ultralong HAP nanowires Figure 3 shows the pH values, ORP values, and numbers of bacterial colony of the fire-resistant paper pulps with different amounts of IA stored at room temperature (28–36 °C) for 30 days in summer. The research findings indicated that the microorganisms could grow in the fire-resistant paper pulp based on ultralong HAP nanowires without IA stored at room temperature (28–36 °C) for 30 days in summer. On the first day, the pH value, ORP, and number of the bacterial colony in the fire-resistant paper pulp based on ultralong HAP nanowires without IA are 7.3, 123 mV, and 987 RLU (relative luminescence units), respectively. After storage of the fire-resistant paper pulp based on ultralong HAP nanowires without IA for 30 days at room temperature in summer, the number of the colony increased significantly to 58,460 RLU, which was 59 times the bacterial colony count of the first day, and the pH value decreased to 6, and ORP was measured to be −376 mV, due to the proliferation of microorganisms, which indicated that the organic acids and reductive metabolites were produced during the reproduction of microorganisms.

The experimental results indicated that the addition of IA could effectively inhibit the reproduction of microorganisms in the fire-resistant paper pulp. The fire-resistant paper pulps with 30 g, 60 g, 90 g, 120 g of IA showed similar and excellent antibacterial performance. The pH value, ORP, and number of the bacterial colony were in the range of 8.1–8.7, 143–191 mV, and 2–5.7 RLU, respectively, and their changes were not obvious for the first day and the 30th day. The experimental results indicated that the fire-resistant paper pulp based on ultralong HAP nanowires in the presence of IA had excellent antibacterial performance, and the antibacterial agents were not necessary in the actual production of the fire-resistant paper based on ultralong HAP nanowires.

**Figure 4:**
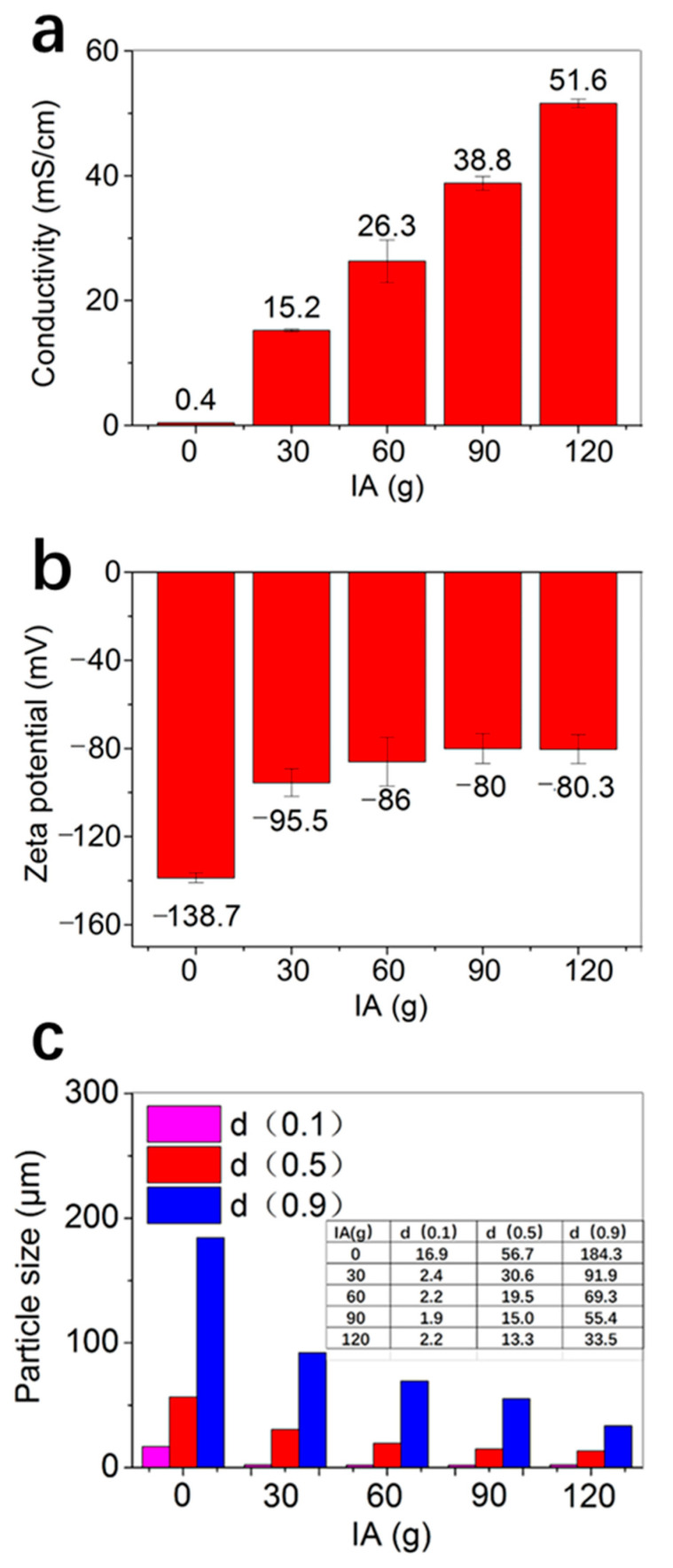
Conductivities (**a**), Zeta potentials (**b**), and particle sizes (**c**) of the fire-resistant paper pulps based on ultralong HAP nanowires with different added amounts of IA.

The experimental results indicated that the flocculation properties of the fire-resistant paper pulp based on ultralong HAP nanowires were affected by the conductivity and Zeta potential. Figure 4 showed the conductivities, Zeta potentials, and particle sizes of the fire-resistant paper pulps based on ultralong HAP nanowires with different added amounts of IA. The conductivity and Zeta potential of the fire-resistant paper pulp without IA are 40 mS/m^−1^ and −138.7 mV, respectively. With the increasing addition amount of IA from 30 to 120 g, the conductivity of the fire-resistant paper pulp increased from 1520 to 5160 mS m^−1^. Attributing to the increased conductivity of the fire-resistant paper pulp, the diffusion double layer on the surface of the floccules was compressed, resulting in a decreased absolute value of Zeta potential, although they were still negative. For example, the absolute value of the Zeta potential decreased from 95.5 to 80.3 mV when the addition amount of IA increased from 30 to 120 g. The Zeta potential was a measure of the degree of mutual repulsion or attraction between particles in aqueous solution. When the absolute value of the Zeta potential exceeded 60 mV, the pulp floccules were very stable, which was also verified by the particle sizes of paper pulp floccules. The particle size corresponding to 50% of the cumulative particle size distribution (d (0.5)) of paper pulp floccules without IA is 56.7 µm, and the particle size of paper pulp floccules with 30 g, 60 g, 90 g, and 120 g IA was 30.6 µm, 19.5 µm, 15 µm, and 13.3 µm, respectively. The above experimental results indicated that the addition of IA could significantly increase the conductivity of the fire-resistant paper pulp based on ultralong HAP nanowires and reduced the particle size of paper pulp floccules.

**Figure 5:**
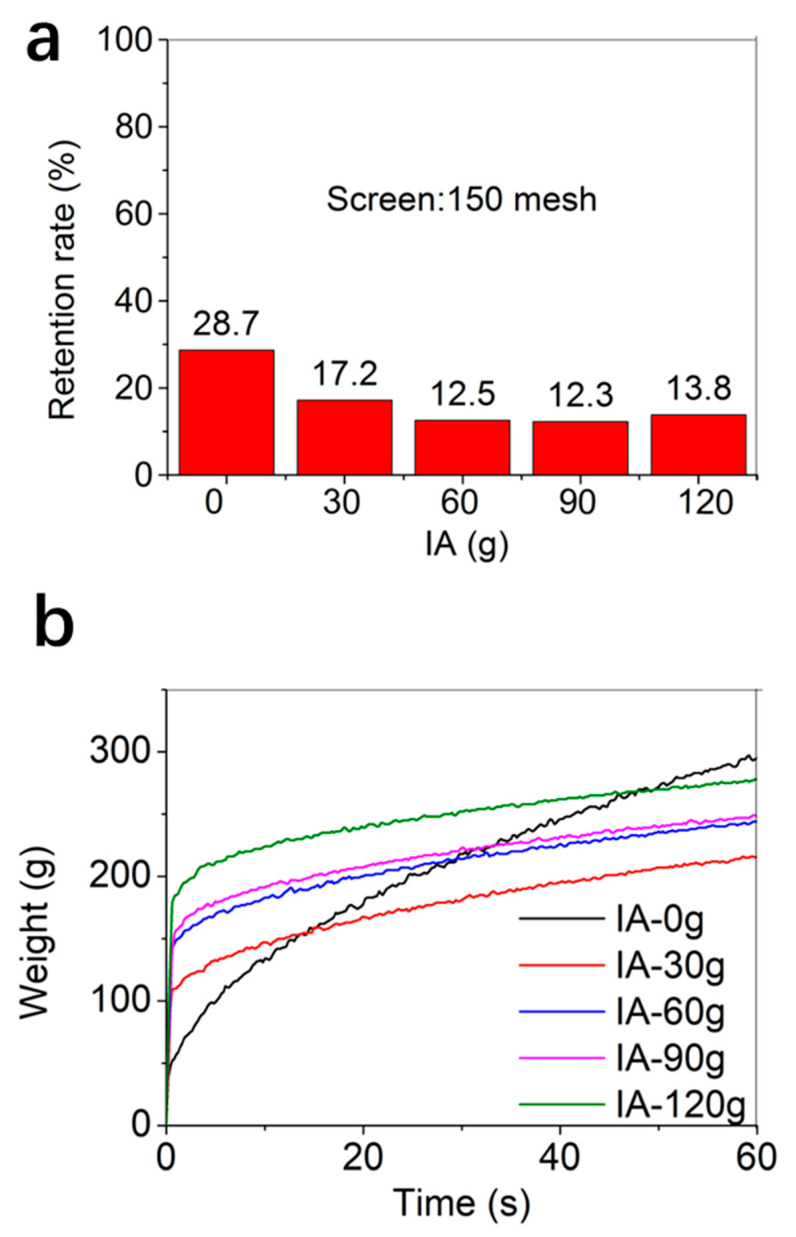
The retention percentages (**a**) and drainability curves (filtered water weight versus filtration time) (**b**) of the fire-resistant paper pulps based on ultralong HAP nanowires with different added amounts of IA.

The wet paper sheet can be formed using the paper pulp by filtration, which means the paper pulp is firstly kept on the web by mechanical interception. In the actual industrial production process of paper, the mesh number or the number of layers is different, which also leads to a difference in the pulp retention rate. In this study, 150-mesh filter is adopted, which is the smallest pore of the forming network for BTG laboratory dynamic water filter. Figure 5 shows the retention percentages and drainabilities of the fire-resistant paper pulps based on ultralong HAP nanowires with different added amounts of IA. The results in Figure 5a indicated that the retention percentage of the fire-resistant paper pulp was low. When the addition amount of IA was 0 g, 30 g, 60 g, 90 g, and 120 g, the retention percentage was 28.7%, 17.2%, 12.5%, 12.3%, 13.8%, respectively. In conclusion, the addition of IA had a negative effect on the retention percentage of the fire-resistant paper pulp based on ultralong HAP nanowires. The drainability and retention performance are equally important in the paper wet end forming process. Since the drainability affects the working speed of the paper machine, and further influences the production efficiency of paper. In addition, the poor drainability will cause the difficulty in the wet paper forming. Figure 5b indicated that the filtered water weight of the fire-resistant paper pulp without IA increased gradually with the filtration time, and the wet paper layer was formed during the filtration process, which further slowed down the filtration rate. After adding IA in the fire-resistant paper pulp, the trend of the drainability curve was obviously different from that in the absence of IA. The weight of filtered water increased rapidly at the early stage, and then increased slowly. In conclusion, the drainability of the fire-resistant paper pulp based on ultralong HAP nanowires was improved with the increased addition amount of IA.

### 2.4. Effects of Wet End Chemical Properties on Performances of the Fire-Resistant Paper

**Figure 6:**
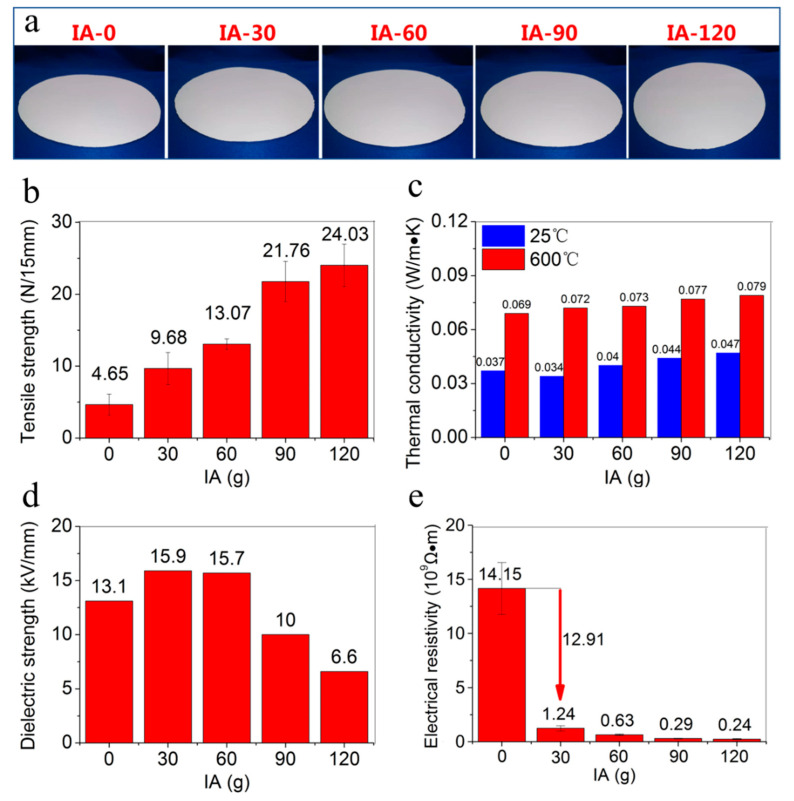
Digital images (**a**), tensile strengths (**b**), thermal conductivities (**c**), dielectric strengths (**d**), electrical resistivities (**e**) of the fire-resistant paper sheets based on ultralong HAP nanowires with different addition amounts of IA.

The wet end chemical properties of the fire-resistant paper pulp based on ultralong HAP nanowires not only affected the retention and filtration properties, but also affected the properties of the as-prepared fire-resistant paper. The appearance of the as-prepared fire-resistant paper based on ultralong HAP nanowires was similar to that of the traditional paper based on cellulose fibers, as shown in Figure 6a. The basic parameters and physical properties of the as-prepared fire-resistant paper based on ultralong HAP nanowires are shown in Table 1 and Figure 6b–e. The basis weight of the as-prepared fire-resistant paper without IA was 92.4 g m^−2^. When the addition amount of IA increased from 30 to 120 g, the basis weight of the as-prepared fire-resistant paper increased from 92.7 to 137.6 g m^−2^, this was due to the nanometer-sized IA particles filling the porous structure and increasing the weight of the fire-resistant paper. The thickness of the as-prepared fire-resistant paper increased with increasing addition amount of IA from 0 to 120 g. The thickness of the as-prepared fire-resistant paper without IA was 128 µm, and the thickness of the fire-resistant paper increased from 139 to 202 µm with increasing addition amount of IA from 30 to 120 g. The addition of IA slightly decreased the tightness of the fire-resistant paper and increases the bulkness of the fire-resistant paper. The tightness and bulkness of the fire-resistant paper without IA are 720 kg m^−3^ and 1.39 × 10^−3^ m^3^ kg^−1^, respectively. After adding 30~120 g of IA, the tightness of the fire-resistant paper was in the range of 670~690 kg m^−3^, and the bulkness of the fire-resistant paper ranged from 1.45 × 10^−3^ to 1.50 × 10^−3^ m^3^ kg^−1^.

Furthermore, the tensile strength of the fire-resistant paper increased with increasing addition amount of IA, as shown in Figure 6b. The tensile strength of the fire-resistant paper without IA was 4.65 N (paper width 15 mm), and the tensile strength of the fire-resistant paper increased from 9.68 to 24.03 N (paper width 15 mm) with increasing amounts of IA from 30 to 120 g, indicating that the IA as a glue could significantly enhance the tensile strength of the fire-resistant paper based on ultralong HAP nanowires.

As a type of inorganic material, the important features of ultralong HAP nanowires included high-temperature resistance, nonflammability, heat insulation, and electrical insulation. The fire-resistant paper based on ultralong HAP nanowires had a promising application prospect in various fields, such as high-temperature resistance, fire retardance, heat insulation, and electrical insulation. The thermal conductivities of the fire-resistant paper without IA were 0.037 W m^−1^ K^−1^ (25 °C) and 0.069 W m^−1^ K^−1^ (600 °C). After the addition of IA, there was a slight increase in thermal conductivity of the fire-resistant paper (Figure 6c).

As shown in Figure 6d, the dielectric strength of the fire-resistant paper without IA was 13.1 kV mm^−1^. The dielectric strength of the fire-resistant paper was 15.9 and 15.7 kV mm^−1^ when the addition amount of IA was 30 and 60 g, respectively, which was higher than that of the fire-resistant paper without IA. However, with further increase in the amount of IA to 90 and 120 g, the dielectric strength of the fire-resistant paper decreased to 10 and 6.6 kV mm^−1^, respectively. The electrical resistivity of the fire-resistant paper dropped dramatically after the addition of IA, as shown in Figure 6e. The electrical resistivity of the fire-resistant paper without IA is 1.415 × 10^10^ Ω m, the value was 1.24 × 10^9^ Ω m after adding 30 g of IA. As the amount of IA further increased, the electrical resistivity of the fire-resistant paper further decreased (~10^8^ Ω m) (Figure 6e). For practical applications, we can choose the appropriate amount of IA according to the performance requirements and cost of the fire-resistant paper based on ultralong HAP nanowires.

## 3. Experimental

### 3.1. Materials and Chemicals

Calcium chloride (CaCl_2_) and sodium dihydrogen phosphate dihydrate (NaH_2_PO_4_·2H_2_O) were purchased from Sinopharm Chemical Reagent Co., Ltd. (Shanghai, China). Sodium oleate was obtained from Shanghai Jinyue Chemical Co., Ltd. (Shanghai, China). Ethanol was purchased from Shanghai Lingfeng Chemical Reagent Co., Ltd. (Shanghai, China). The inorganic adhesive (IA) was prepared by the authors’ laboratory. All chemicals except sodium oleate were analytical reagent grade; sodium oleate was industrial grade. All chemicals were used as received without further purification. Deionized water was used in all related experiments.

### 3.2. Synthesis of Ultralong HAP Nanowires

Ultralong HAP nanowires were synthesized according to the calcium oleate precursor hydrothermal method previously reported by the author’s research group [25,31]. In this method, sodium oleate was used as a reactant and a surfactant, CaCl_2_ was used as the calcium source, NaH_2_PO_4_·2H_2_O was adopted as the phosphorus source, and deionized water was used as the only solvent. The hydrothermal reaction was performed in a stainless steel autoclave with a volume of 10 L at 200 °C for 36 h. The hydrothermal product was washed with ethanol and deionized water three times, respectively.

### 3.3. Preparation of the Fire-Resistant Paper Pulp and Fire-Resistant Paper

The preparation process of the fire-resistant paper pulp and fire-resistant paper was similar to that of the traditional paper making, including pulping, vacuum filtration, pressing, and drying. First, glass fibers were dispersed in tap water under stirring. Then, an aqueous suspension containing ultralong HAP nanowires and an aqueous suspension of homemade IA were added into the above aqueous suspension, respectively. The fire-resistant paper pulps with different weights of IA aqueous suspensions ranging from 30–120 g were obtained. The fire-resistant paper was prepared using a sheet former, then pressing (4 MPa, 3 min), and drying (105 °C, 3 min).

### 3.4. Characterization

The samples were characterized by scanning electron microscopy (SEM, Magellan 400, FEI, Hillsboro, Oregon, USA; Hitachi TM3000, Tokyo, Japan). Thermogravimetric (TG) analysis was performed using a thermal analyzer (STA 449/PC, Netzsch, Selb, Germany) with a heating rate of 10 °C min^−1^ in flowing air. The pH value, conductivity, oxidation-reduction potential (ORP), microbial count, Zeta potential, and particle size were measured with a pH meter, conductivity meter, ORP meter, 3M ATP fluorimeter, Zeta potentiometer, and particle size analyzer (Malvern Mastersizer 2000), respectively. The pulp retention and filtration performance tests were measured with BTG dynamic water filter. The thermal conductivity was measured with a thermal conductivity analyzer (Hot Disk TPS 2500S, Sweden). The tensile tests were carried out using a universal testing machine (DRK-101, Drick, Jinan, China). Dielectric strengths were measured in air using a voltage tester (YD2665, Changzhou Yangzi Electronic Co., Ltd., Changzhou, China) under stepwise increasing voltage. The electrical resistivity values were measured with the precision impedance analyzer (Agilent 4294A). The physical properties of the fire-resistant paper were measured according to the Technical Association of the Pulp and Paper Industry (TAPPI) standards.

## 4. Conclusions

In this work, the wet end chemical properties of a new kind of the fire-resistant paper pulp based on ultralong HAP nanowires are studied. The inorganic adhesive and its addition amount have important influence on the wet end chemical parameters (pH/conductivity/ORP/Zeta potential/particle size), which affect the runnability of the paper machine and the properties of the as-prepared fire-resistant paper. The experimental results indicated that the fire-resistant paper pulp based on ultralong HAP nanowires in the presence of IA possesses excellent antibacterial performance, even after being stored in the environment (28–36 °C) in summer for one month. The pH value and ORP of the fire-resistant paper pulp based on ultralong HAP nanowires were relatively stable in the range of 8.1–8.7 and 143–191 mV, respectively, indicating that bacteria do not proliferate obviously. The flocculation properties of the fire-resistant paper pulp based on ultralong HAP nanowires were affected by the conductivity and the Zeta potential. The conductivity of the fire-resistant paper pulp increased and the absolute value of Zeta potential exceeds 60 mV, and particle size d (0.5) of paper pulp floccules was <50 µm after the addition of IA, which resulted in the low retention percentage of the fire-resistant paper pulp. In addition, the addition of IA could increase the tensile strength of the fire-resistant paper based on ultralong HAP nanowires. When the addition amount of IA is 60 g, the tensile strength, dielectric strength, thermal conductivity, the electrical resistivity of the fire-resistant paper was 13.07 N (15 mm width), 15.7 kV mm^−1^, 0.04 W m^−1^ K^−1^, 6.3 × 10^8^ Ω m, respectively. The fire-resistant paper based on ultralong HAP nanowires had a promising application prospect in various fields, such as high-temperature resistance, fire retardance, heat insulation, and electrical insulation. For practical applications, we can choose the appropriate amount of IA according to the performance requirements and cost of the fire-resistant paper.

## Figures and Tables

**Table 1:** Physical properties of the as-prepared fire-resistant paper sheets based on ultralong HAP nanowires with different addition amounts of IA.

IA (g)	Basis Weight (g m^−2^)	Thickness (µm)	Tightness (g cm^−3^)	Bulkness (cm^3^ g^−1^)
0	92.4	128	0.72	1.39
30	92.7	139	0.67	1.50
60	112.4	166	0.68	1.48
90	124.8	181	0.69	1.45
120	137.6	202	0.68	1.47

## Data Availability

The data presented in this study are available on request from the corresponding author.

## Sample Availability

Samples are available from the authors.
